# Radiofrequency echographic multi-spectrometry for the in-vivo assessment of bone strength: state of the art—outcomes of an expert consensus meeting organized by the European Society for Clinical and Economic Aspects of Osteoporosis, Osteoarthritis and Musculoskeletal Diseases (ESCEO)

**DOI:** 10.1007/s40520-019-01294-4

**Published:** 2019-08-17

**Authors:** Adolfo Diez-Perez, Maria Luisa Brandi, Nasser Al-Daghri, Jaime C. Branco, Olivier Bruyère, Loredana Cavalli, Cyrus Cooper, Bernard Cortet, Bess Dawson-Hughes, Hans Peter Dimai, Stefano Gonnelli, Peyman Hadji, Philippe Halbout, Jean-Marc Kaufman, Andreas Kurth, Medea Locquet, Stefania Maggi, Radmila Matijevic, Jean-Yves Reginster, René Rizzoli, Thomas Thierry

**Affiliations:** 1 Department of Internal Medicine, Hospital del Mar/IMIM and CIBERFES, Autonomous University of Barcelona, Passeig Maritim 25-29, 08003 Barcelona, Spain; 2 FirmoLab Fondazione F.I.R.M.O., Florence, Italy; 3 Department of Biological, Experimental and Clinical Science, University of Florence, Florence, Italy; 4 Chair for Biomarkers of Chronic Diseases, Biochemistry Department, College of Science, King Saud University, Riyadh, Kingdom of Saudi Arabia; 5 NOVA Medical School, Universidade Nova de Lisboa, Lisbon, Portugal; 6 WHO Collaborating Centre for Public Health Aspects of Musculoskeletal Health and Aging, University of Liège, Liège, Belgium; 7 MRC Lifecourse Epidemiology Unit, Southampton General Hospital, University of Southampton, Southampton, UK; 8 Department of Rheumatology and EA 4490, University-Hospital of Lille, Lille, France; 9 Bone Metabolism Laboratory, Jean Mayer USDA Human Nutrition Research Center on Aging, Tufts University, Boston, MA USA; 10 Department of Internal Medicine, Division of Endocrinology and Diabetology, Medical University of Graz, Graz, Austria; 11 Department of Medicine, Surgery and Neurosciences, University of Siena, Siena, Italy; 12 Frankfurter Hormon und Osteoporose Zentrum, Frankfurt, Germany; 13 International Osteoporosis Foundation, Nyon, Switzerland; 14 Department of Endocrinology, Ghent University Hospital, Ghent, Belgium; 15 Department of Orthopaedic Surgery and Osteology, Klinikum Frankfurt, Frankfurt, Germany; 16 Mayor Teaching Hospital, Charite Medical School, Berlin, Germany; 17 Department of Public Health, Epidemiology and Health Economics, University of Liège, Liège, Belgium; 18 National Research Council, Aging Program, Institute of Neuroscience, Padua, Italy; 19 Faculty of Medicine, University of Novi Sad, Novi Sad, Serbia; 20 Clinical Center of Vojvodina, Clinic for Orthopedic Surgery, Novi Sad, Serbia; 21 Service of Bone Diseases, Geneva University Hospitals and Faculty of Medicine, Geneva, Switzerland; 22 Department of Rheumatology, Hospital Nord, CHU St Etienne, St Etienne, France; 23 INSERM 1059, University of Lyon, St Etienne, France

**Keywords:** Bone strength assessment, Osteoporosis diagnosis, Fracture risk, REMS, Ultrasound, Lumbar spine, Femoral neck

## Abstract

**Purpose:**

The purpose of this paper was to review the available approaches for bone strength assessment, osteoporosis diagnosis and fracture risk prediction, and to provide insights into radiofrequency echographic multi spectrometry (REMS), a non-ionizing axial skeleton technique.

**Methods:**

A working group convened by the European Society for Clinical and Economic Aspects of Osteoporosis and Osteoarthritis met to review the current image-based methods for bone strength assessment and fracture risk estimation, and to discuss the clinical perspectives of REMS.

**Results:**

Areal bone mineral density (BMD) measured by dual-energy X-ray absorptiometry (DXA) is the consolidated indicator for osteoporosis diagnosis and fracture risk assessment. A more reliable fracture risk estimation would actually require an improved assessment of bone strength, integrating also bone quality information. Several different approaches have been proposed, including additional DXA-based parameters, quantitative computed tomography, and quantitative ultrasound. Although each of them showed a somewhat improved clinical performance, none satisfied all the requirements for a widespread routine employment, which was typically hindered by unclear clinical usefulness, radiation doses, limited accessibility, or inapplicability to spine and hip, therefore leaving several clinical needs still unmet. REMS is a clinically available technology for osteoporosis diagnosis and fracture risk assessment through the estimation of BMD on the axial skeleton reference sites. Its automatic processing of unfiltered ultrasound signals provides accurate BMD values in view of fracture risk assessment.

**Conclusions:**

New approaches for improved bone strength and fracture risk estimations are needed for a better management of osteoporotic patients. In this context, REMS represents a valuable approach for osteoporosis diagnosis and fracture risk prediction.

## Introduction

Osteoporosis is a systemic skeletal disease characterised by the reduction in bone mass and the degeneration of bone structure that leads to an increased risk of fracture [1], with important social, medical and community care costs.

**Fig. 1:**
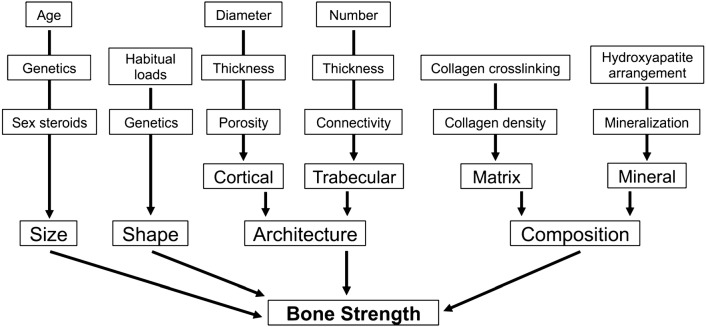
Factors contributing to bone strength. The ultimate definition of bone strength is complex, but four main categories of bone characteristics that contribute to bone strength can be outlined at different scale level: size, shape, architecture and composition [3]

Bone strength, which is a measure of the resistance to bone fracture [2], is determined by a composite summation of numerous skeletal characteristics that can be divided into four basic components: composition, microarchitecture, size, and shape (Fig. 1). Several factors contribute to the definition of each skeletal characteristic, such as cellular density, mineralization, collagen crosslinking at a nanoscale level for the definition of bone composition; trabecular and cortical properties including porosity, thickness, connectivity at microscale level for the definition of bone microarchitecture; age, genetics, gender, and habits for the definition of bone size and shape at a macroscale level. Understanding how these variables interact and contribute to bone strength is critical in the development of fracture prediction tools [3, 4].

Available imaging techniques are able to capture a measure of the aforementioned factors and could also provide information about bone strength. Therefore, in principle, several tools are necessary to assess the different bone properties, such as spectroscopy and microscopy at nano-scale level, high-resolution quantitative computed tomography (HR-QCT) or µCT at micro-scale level, dual-energy X-ray absorptiometry (DXA) or CT at macro-scale level [3, 4]. Nowadays, in clinical practice, bone strength is indirectly estimated by bone mineral density (BMD) measured by DXA, which currently represents a well-recognized technology for the diagnosis of osteoporosis worldwide. Nonetheless, several studies demonstrated that BMD only accounts for about 50–70% of the bone strength variation, being able to describe features related to the quantity of bone tissue but overlooking information about bone quality [4–6].

These considerations argue for the introduction in clinical routine of further tools in order to better define and estimate bone strength and to predict the risk of fracture.

To address this, a working group convened by the European Society of Clinical and Economic Aspects of Osteoporosis and Osteoarthritis (ESCEO) met to review the data on the currently available imaging techniques for the in vivo bone strength assessment and to discuss principles, applications and perspectives of a new technology called Radiofrequency Echographic Multi-Spectrometry (REMS) on the basis of the latest available data. Specific attention has been paid to identify the clinical needs related to bone strength assessment, fracture risk prediction and osteoporotic patient management that are not completely satisfied by the techniques currently employed in clinical routine and to their possible resolution by the introduction of innovative approaches.

## Clinical assessment of bone strength

Several approaches have been developed to provide an estimation of bone strength, including imaging devices, risk fracture calculators, bone biopsy techniques, and laboratory tests (Table 1). In this review, we will focus on the imaging devices: the main features of the most common approaches are described and discussed in the following paragraphs.

**Table 1:** Main currently available tools for the in vivo assessment of bone strength

Category	Tool
Imaging devices	Dual X-ray absorptiometry (DXA)
	Quantitative CT (QCT)
	High resolution peripheral QCT (HR-pQCT)
	Magnetic resonance imaging (MRI)
	Quantitative ultrasound (QUS)
	Radiofrequency echographic multi spectrometry (REMS)
Risk fracture calculators	FRAX^®^
	Qfracture^®^
	Garvan^®^
	DVO risk calculator^®^
Bone biopsy techniques	Static histomorphometry
	Dynamic histomorphometry
Laboratory tests	Bone turnover markers (BTMs)
	Single nucleotide polymorphisms (SNPs)
	Micro-ribonucleic acid (miRNA)
Others	Microindentation

### Dual-energy X-ray absorptiometry

The most widely used technique to obtain information about bone strength is DXA, currently considered as the gold standard densitometric technique for the diagnosis of osteoporosis. Through the acquisition of two-dimensional (2D) X-ray scans with an effective dose in the range of 1–13 µSv [7–9], it estimates an areal BMD expressed in g/cm^2^ (i.e., the ratio between bone mineral content estimated through X-ray absorption and 2D projection of the scanned bone area), which is a surrogate measure of bone strength [3]. Ex vivo studies performed on isolated human bones showed that areal BMD accounts for about two thirds of the variation of bone strength as evaluated by shear test of the femoral neck or compression of the vertebrae [5, 10].

In clinical practice, DXA is employed to categorise the patient by comparing the measured BMD value with the average BMD of a reference healthy population of young individuals, which allows to define the patient’s *T*-score as the number of standard deviations (SDs); the measured BMD differs from the reference value. Thus, patients are classified as healthy (normal BMD, *T*-score ≥ − 1), osteopenic (− 2.5 < *T*-score < − 1), osteoporotic (*T*-score ≤ − 2.5) or severely osteoporotic (*T*-score ≤ − 2.5 in the presence of one or more fragility fractures), according to the definitions given by the World Health Organization (WHO) [11, 12]. Moreover, DXA is employed to provide prognostic information on probability of fracture and to monitor bone status in longitudinal studies [13]. A meta-analysis of prospective cohort studies correlating the baseline BMD with the subsequent follow-up for fractures showed that the measurement at the spine and at the hip seemed to have a better predictive ability for spine and hip fractures, respectively [14]. Leslie et al. investigating whether the rate of BMD loss would predict fracture risk independently of current BMD, found that routine clinical DXA measurement in untreated women cannot accurately characterise the rate of BMD loss due to the dominating effect of measurement errors [15].

BMD alone for the assessment of fracture risk has a high specificity but low sensitivity, with many fragility fractures occurring in patients who did not have a diagnosis for osteoporosis as defined by the WHO classification based on *T*-score value, but whose BMD lay in the osteopenic range [16–18]. Moreover, in the diabetes paradigm, BMD is unable to explain the increased risk of fracture: in particular, for Type 1 diabetes, the highly increased risk of hip fracture was only partially explained by the observed BMD reduction, whereas in Type 2 diabetes the increased risk of fracture is not captured by the paradoxically higher BMD [19].

Thus, a screening program for osteoporosis based on DXA-measured BMD alone cannot be recommended for the whole population [20, 21], and further studies evaluating the most cost-effective screening strategy are warranted [22]. These findings also suggest the hypothesis that multiple factors, other than BMD, contribute to fracture risk and highlight the importance of BMD-independent determinants of bone strength and fracture risk assessment.

DXA scanners, if equipped with dedicated software modules, can also measure additional parameters related to textural features and hip geometry, as described in the subsequent paragraphs.

#### Trabecular bone score

The Trabecular Bone Score (TBS), calculated by the software package TBS iNsight^®^ (Medimaps Group SA, Geneva, Switzerland), is a unit-less indirect index of trabecular microarchitecture based on pixel grey level variations in the DXA images [23, 24]. The large clinical database of the Province of Manitoba (Canada), including 29,407 women aged over 50 years at baseline, has been used for several investigations aimed at assessing the ability of TBS in fracture risk prediction. Although TBS (along with BMD and additional clinical risk factors) helps to identify individuals at high risk of fracture and to guide initiation of osteoporosis treatment [25], change in lumbar spine TBS is not a useful indicator of fracture risk regardless of osteoporosis treatment [26]. Another retrospective study on the Manitoba database showed the ability of lumbar spine TBS to predict osteoporotic fractures in patients with type 2 diabetes, whereas BMD was paradoxically increased in these patients [27].

TBS values can be also input into the Fracture Risk Assessment tool FRAX^®^ [28]. This tool, available online at www.shef.ac.uk/FRAX [29], aims to predict osteoporotic fractures on the basis of several risk factors optionally including femoral neck BMD or spinal TBS [17]. A recent meta-analysis showed that TBS, adjusted for time since baseline measurement and age, has a significant correlation with major osteoporotic fractures, which is slightly improved when FRAX^®^ probability is taken into account, supporting the use of TBS both as a stand-alone fracture risk prediction tool and as a valuable add-on to FRAX^®^ [30].

Nonetheless, some limitations of TBS should be also mentioned: increased noise in DXA images results in an artefactually reduced TBS [31]; TBS, but not BMD, is dependent on the used scan mode (standard versus thick), at least for GE Lunar Prodigy scanners [32]; increases in soft-tissue thickness overlying the spine lead to diminished TBS [33]; TBS reliability in men depends on BMI [34, 35].

Furthermore, a debate is still open in the scientific literature as regards the proportion in which TBS explains the variability in vertebral strength. The very few available biomechanical studies [36, 37], which anyway represent only an indirect approach to what happens in vivo, found poor or no direct correlation between TBS and vertebral failure load, although reporting in some cases significant correlations with other elastic properties (e.g., *r *= 0.64 with bone stiffness [36]).

Overall, lumbar spine TBS is an evolving software that has been shown to be useful as a complementary fracture risk prediction tool. However, the magnitude of TBS increase in osteoporosis treatment is smaller than that of BMD. The relationship between change in TBS and fracture risk reduction remains to be elucidated with recent and ongoing studies that are helping to refine its clinical utility [28, 38].

#### Hip axis length and hip structural analysis

**Fig. 2:**
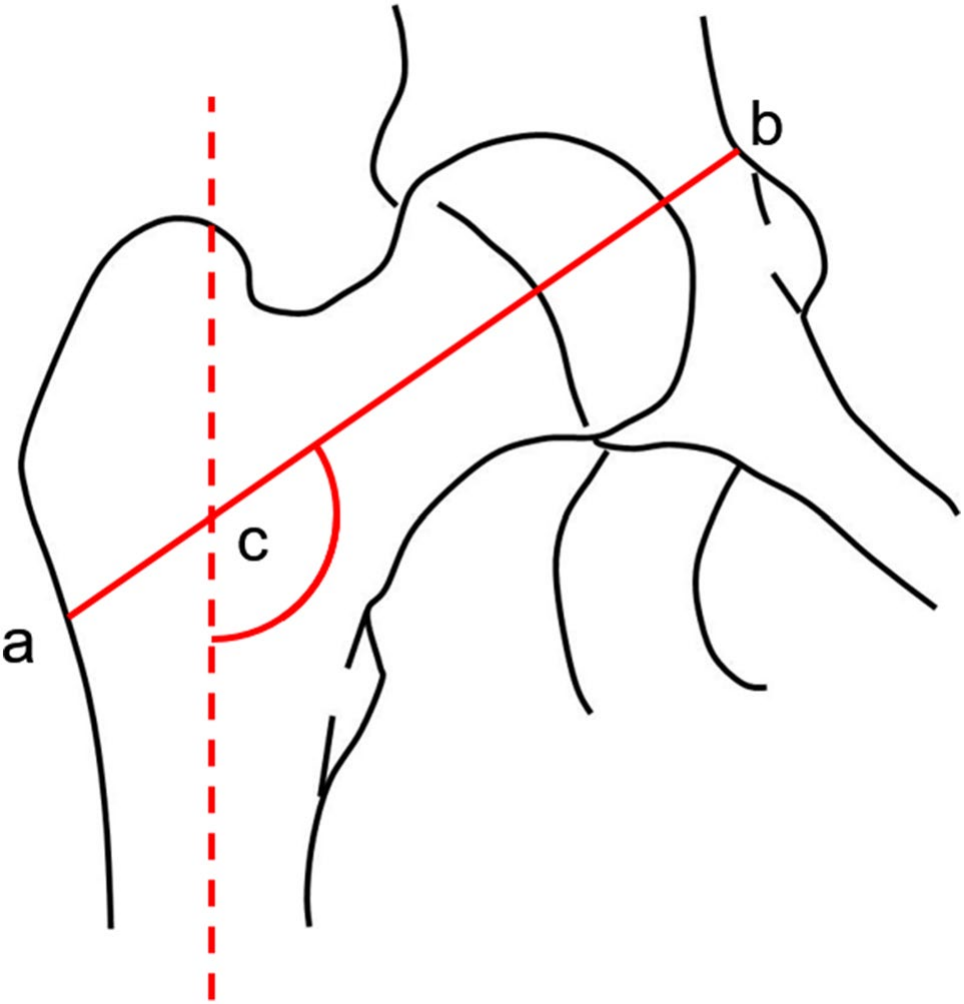
Representation of the Hip Axis Length (HAL) definition, i.e. the distance from the base of the greater trochanter to the inner pelvic brim (segment a–b). Angle c is the neck shaft angle, i.e. the angle between the derived axes of the femoral neck and shaft

The hip axis length (HAL), i.e. the distance from the base of the greater trochanter to the inner pelvic rim (Fig. 2), is a further non-BMD parameter that has been considered for the assessment of hip bone strength through DXA [39]. A study by Leslie et al. [40] showed that the risk of hip fracture, adjusted for age and femoral neck BMD, increases by 3.6% in men (*p* = 0.022) and by 4.6% in women (*p* < 0.001) for every millimetre of increase in HAL. However, as pointed out by the International Society of Clinical Densitometry (ISCD) at the 2015 Position Development Conference, the association between HAL and hip fracture is actually ambiguous, with 14 published studies showing a positive association between HAL and hip fracture, 11 studies showing no association and 1 study reporting a negative association [41]. On this basis, the Hip Structural Analysis (HSA) software was developed to simultaneously evaluate both hip geometrical information and corresponding mineral mass data derived from DXA images [42]. Whether HSA could be actually useful for clinical assessment of bone fragility is currently under investigation, with few studies showing that some factors, i.e. cortical thickness of the intertrochanteric region [43], HAL and neck shaft angle [44] might have a role in fracture risk assessment for specific categories of patients.

### Quantitative computed tomography

Quantitative computed tomography (QCT) allows to obtain a tomographic 3D reconstruction of the scanned bone and provides a volumetric bone mineral density (vBMD), expressed in g/cm^3^, which is less susceptible to osteo-arthritis artefacts than the areal BMD measured by DXA. Depending on the specific adopted protocol, the exposure dose for a lumbar QCT scan is in the range of 50–500 µSv, which is clearly lower than a standard abdominal CT scan (approximatively 8 mSv) [7, 8]. In contrast to DXA, this analysis allows separate estimation of trabecular and cortical BMD. Since trabecular bone has a substantially higher metabolic turnover than cortical bone, QCT is more sensitive to changes in BMD than DXA [45]. The American College of Radiology (ACR) has recently introduced practice guidelines for the use of QCT, with the definition of equivalent WHO classification of osteoporosis classes based on spine trabecular volumetric BMD measurements and an association between volumetric BMD and risk of fracture has also been established. Moreover, the correlation between BMD measured by QCT and derived by DXA has also been assessed. Thus, QCT might be used in case of non-availability of DXA. It has also some important advantages over DXA that are useful in specific clinical indications, i.e. abnormal body size (small/large body frame) or very obese patients, advanced degenerative spine disease (e.g. idiopathic skeletal hyperostosis, degenerative disc disease, facet arthropathy), and need for accurate monitoring of trabecular metabolic activity [46].

In addition, QCT allows the development of finite-element analysis (FEA), a computational method in which each voxel represents a finite element with assigned material properties (e.g., elastic modulus). Load simulations might be performed with the estimation of the load at which structural failure occurs, so identifying a biomechanical fracture threshold [47]. Indeed, the prospective 5-year AGES-Reykjavik case–control study of 1110 patients reported significant correlation between bone strength estimated by FEA and fracture occurrence, with age-adjusted odds ratios for low vertebral strength and incident spine fractures of 2.8 (95% CI 1.8–4.3) and 2.2 (2.2, 95% CI 1.5–3.2), and age-adjusted odds ratios for low femoral strength and incident hip fractures of 4.2 (95% CI 2.6–6.9) and 3.5 (95% CI 2.3–5.3) for women and men, respectively [48]. The FEA performed on QCT images also provides information on femoral strength changes associated with therapies, as in the “PaTH” study evaluating the effects of PTH(1–84) and/or alendronate in osteoporotic women [49]. It was shown that strength was mainly influenced by trabecular density changes, regardless of the pharmacological treatment, although changes in the cortical bone density and overall bone geometry also contributed to femoral strength changes, in that case depending on the drug used. Due to the limited clinical availability of QCT and to the associated high costs and radiation doses [50], in the past years, several studies investigated the possibility to obtain finite-element models from DXA imaging, but their clinical feasibility remains very limited and their actual usefulness should be further explored [51–53].

Peripheral QCT (pQCT) has a more widespread availability and examines appendicular skeleton sites with a lower effective radiation dose than central QCT (lower than 0.01 mSv [8]). Through the simultaneous calculation of BMD, bone and muscle geometrical parameters, and biomechanical parameters, it provides an evaluation of the “muscle-bone unit”, with the potential of representing a valid functional approach for bone health assessment [54]. However, with respect to DXA, pQCT has anyway a more limited accessibility, being also sensitive to motion artefacts.

### High-resolution peripheral QCT

High-resolution peripheral QCT (HR-pQCT), with a single scan exposure dose of about 3–4 µSv [55, 56], allows one to obtain volumetric images of distal bones, i.e. radius and tibia, with a simultaneous measure of cortical and trabecular vBMD and a deeper representation of bone microarchitecture, thus performing the so-called virtual biopsy. A micro-FEA for peripheral bones might be thus derived from HR-pQCT scans. A cross-section of the distal radius or tibia is scanned at a resolution sufficiently high to resolve the trabecular microstructure (in the order of 80 μm). Based on these images, micro-FE models are generated and a mechanical test is simulated to derive the stiffness of the scanned region and to estimate the strength of the whole bone. In validation studies, micro-FEA showed its ability to predict bone failure load better than any density-based parameter, but this improved strength prediction did not result in a better separation of subjects with and without distal radius fractures in retrospective studies, probably due to the reduced reproducibility, and consequently reduced specificity and sensitivity, in population retrospective studies with respect to validation studies performed on cadavers [57]. Currently, several HR-pQCT devices are available worldwide [58, 59] and their use is mainly reserved for research purposes [60, 61], although there is potential to use this technique in the clinical diagnosis and management of osteoporosis irrespectiwhether its ability in fracture prediction will be fully demonstrated [59, 61].

### Magnetic resonance imaging

In magnetic resonance imaging (MRI), and high-resolution MRI as well, the hydrogen atoms in water molecules are exploited to obtain a contrast between marrow signal (hyper-intense) and trabecular structure (hypo-intense). With this non-ionizing imaging modality, structural trabecular parameters (i.e., trabecular number, thickness, connectivity, anisotropy and shape) might be obtained and FEA can be applied similarly to the case of QCT [62]. However, some important limitations such as high costs, long scan time, motion artefacts and partial volume effects (in case of insufficient image resolution that results in reduced contrast between adjacent tissues and blurring effects) make this imaging technique scarcely employed and investigated in osteoporosis diagnosis [63]. Furthermore, to the best of our knowledge, no significant longitudinal studies have been conducted to assess fracture risk prediction through MRI.

### Quantitative ultrasound

Quantitative ultrasound (QUS) devices measure bone properties using differential reflections and attenuation of pulsed ultrasound waves. According to the ISCD Official Positions, the only validated skeletal site for the clinical use of QUS in osteoporosis management is the heel, which, in conjunction with clinical risk factors, can be used in patients aged over 65 in order to identify low-risk populations [64, 65]. The most common parameters of interest for this non-ionizing technique are those derived from the combination of the speed of sound (SOS) and the broadband ultrasound attenuation (BUA), like the stiffness index (SI) and the quantitative ultrasound index (QUI). SOS, which relies on the accurate measurement of the time employed by a sound wave to pass through the heel, is directly proportional to the BMD. Analogously, BUA, which measures the reduction in intensity at different frequencies of a broadband ultrasound pulse sent through the bone, shows a greater attenuation of the higher frequencies in strong bones than in weak bones [66]. The SI, for instance, was empirically derived as the sum of the normalized BUA and SOS values, with a comparable contribution from the two factors [67].

Population studies have shown the capabilities of heel QUS to predict osteoporotic fractures, especially for hip, with similar sensitivity but lower specificity than DXA in discriminating fractured from non-fractured subjects [68–71]. A meta-analysis on 14 prospective studies showed that the estimated relative fracture risk ranged between 1.23 and 1.94 for each SD decrease in QUS measurements depending on the measured QUS parameter and the type of fracture, suggesting that heel QUS might be used as an alternative to DXA in the assessment of non-vertebral fracture risk [72]. However, it has been also reported that QUS predictive value wanes with time, being for instance more reliable at 1 year after baseline measurement than at 5 years [73].

The main advantages of QUS with respect to DXA are lower cost, smaller space required, portability and absence of ionizing radiation. However, QUS approaches are applicable only to peripheral skeletal sites (calcaneus, tibia, phalanges, radius), and their diagnostic performances are typically inferior to the corresponding DXA ones. Moreover, the availability of several QUS devices that differ substantially from each other in terms of measured parameters has limited their acceptance. Furthermore, QUS results are also dependent on operator, anatomical location, and relative positioning of bone and ultrasound transducer: these drawbacks currently limit the employment of QUS as a clinical diagnostic tool, except for screening purposes [47, 64].

## Role of bone strength in the assessment of fracture risk: state of the art

### Areal BMD

Modelling bone strength might have implications for the understanding of osteoporosis mechanism, the assessment of fracture risk and the prediction, and monitoring of drug treatments. As expressed above, the main clinically available indicator to predict fracture risk is areal BMD estimated by DXA imaging.

In a study by Kopperdahl et al. [48], derived from the AGES-Reykjavik case-control cohort, the large majority of fracture events seems to occur when femoral neck *T*-score of areal BMD was below about − 1.5. However, the ability of this parameter to predict hip fractures significantly improved only if combined with femoral strength estimation for a net reclassification improvement of 33%. Bone strength estimated from QCT-derived FEA was significantly correlated with fracture risk, when adjusted for age and sex, independently from baseline BMD, indicating that a reduced bone strength might be a predictive factor for high risk of osteoporotic fracture.

The predictive ability over a long period of areal BMD was evaluated in the Study of Osteoporotic Fractures (SOF) [74], which assessed BMD and risk factors in almost 8000 women between 1988 and 1990, with a clinical follow-up for fractures lasting for 25 and 20 years for hip and any non-vertebral fracture, respectively. Although BMD alone might be predictive of fracture risk, the overall prediction would be improved by taking into account other risk factors and other bone quality descriptors more closely related to bone strength.

**Fig. 3:**
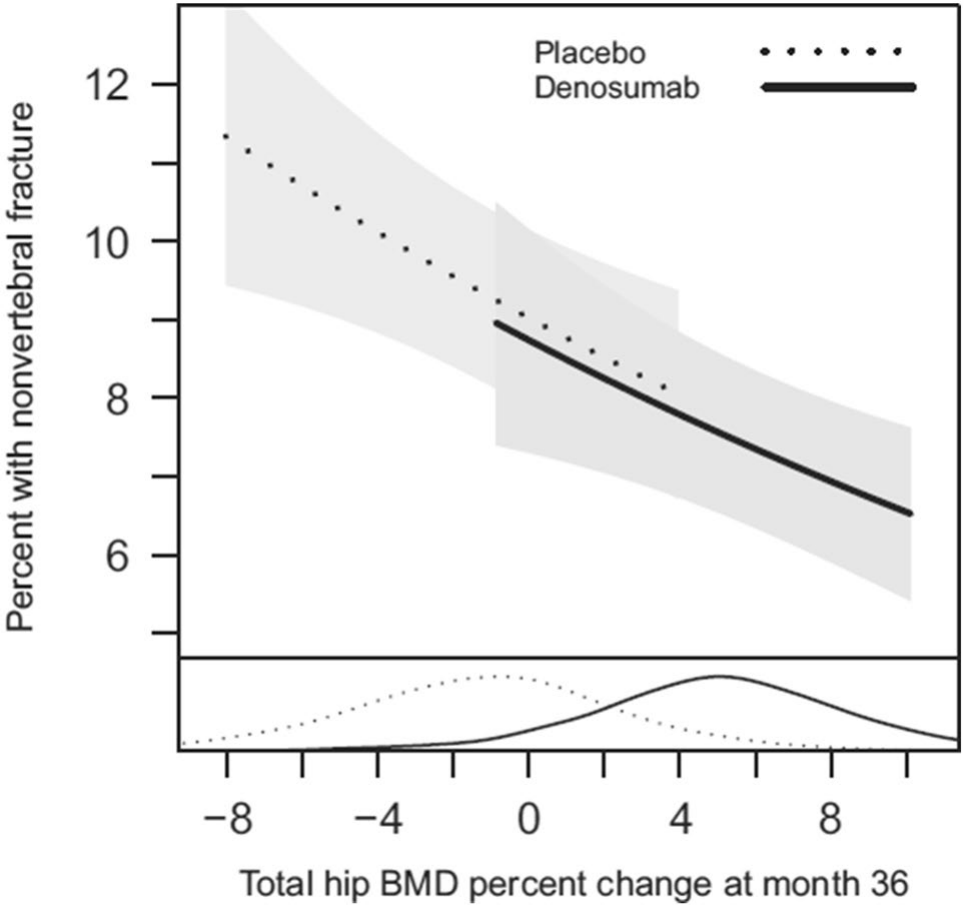
Association between incidence of non-vertebral fracture and total hip BMD percent change from baseline at 36 months in Denosumab and placebo cohorts. The risk of non-vertebral fracture decreased with increasing per cent change in total hip BMD with similar relationships (slopes) for both treatment groups. The density curves at the bottom represent the distributions of total hip BMD change at 36 months for each treatment group [75]

The sensitivity of areal BMD to treatment changes was assessed in studies evaluating drug treatments, such as the FREEDOM study (Denosumab versus placebo) [75] and the HORIZON study (Zoledronic versus placebo) [76], showing that an increase in total hip BMD after 3 years of treatment was correlated with a decrease in risk of non-vertebral fracture and, interestingly, the risk of fracture showed similar relationships (slopes) with per cent change in total hip BMD for both drug and placebo groups (Fig. 3, [75]). Moreover, a meta-regression of 38 placebo-controlled trials with follow-up ranging from 1 to 8 years showed that increments of 2–6% in total hip BMD were significantly associated with a 28–66% reduction in vertebral fractures, as well as with a 16–40% reduction in hip fractures, but not with reductions in non-vertebral fractures [77]. In general, in the osteoporosis treatment, the time-interval of repeated DXA-BMD measurements must be long enough to discern real BMD increments from precision error of repeated measurements (in the order of 1–2%) [78], with reasonable interval not less than 2 years.

In summary, areal BMD estimated by DXA imaging showed reasonable diagnostic performances being discriminative, predictive and sensitive to treatment changes. However, it is widely accepted that a deeper analysis of bone biomechanics is needed to increase the accuracy of fracture risk prediction by taking into account the actual bone strength [3, 17, 79, 80].

### High-resolution peripheral QCT (HR-pQCT)

The main clinical issue associated with the use of BMD alone is the twilight zone of osteopenic patients, where the majority of fractures occurs, suggesting that the standard classification may overlook some important biomechanical characteristics that could improve the assessment of fracture risk [81]. For instance, the discriminative value of parameters derived from HR-pQCT at the distal radius (i.e., total and trabecular density and heterogeneity of trabecular network) was assessed in a setting of osteopenic postmenopausal women (PMW), with and without fractures, in which BMD of the lumbar spine and femoral neck did not significantly differ [82]. Statistically significant differences were found between fractured and non-fractured subjects, with the former having − 10% in total density, − 12.3% in trabecular density, and +25.6% in heterogeneity of trabecular network [82]. This paper, on the one hand, underlined the importance of the description of trabecular microarchitecture of peripheral sites for the enhancement of the prediction of fracture risk in osteopenic patients, but, on the other hand, given the preliminary nature of the study and the small number of enrolled patients, it also emphasized the need of additional studies to better understand the actual usefulness of the mentioned bone microarchitecture parameters. A recent study by the Bone Microarchitecture International Consortium (BoMIC) [83], including 8 large cohorts of patients for a total of 7254 individuals, investigated the association between indices derived from HR-pQCT and fracture risk. During an average follow-up of 4.6 years, 86% of incident fractures were registered in patients with femoral neck BMD in the osteopenic or normal range, but in whom deficits in bone microstructure were found with HR-pQCT. The results indicate that trabecular and cortical bone density and microstructure, as well as estimated failure load measured at the peripheral skeleton, predicted incident fractures independently of femoral neck BMD and FRAX^®^.

Moreover, a large multimodal investigation was performed by Biver et al. [60] in order to assess the contribution of bone phenotype parameters to the prediction of low-trauma fractures in PMW: peripheral trabecular and cortical volumetric BMD and microstructure, as well as the estimated bone strength by HR-pQCT, showed high prediction rate independently of femoral neck BMD, FRAX^®^, and TBS. As BMD assessed by DXA at the ultra-distal radius [84] showed very good performance for fracture prediction, the authors suggested that measuring ultra-distal radius BMD in addition to femoral neck BMD could be useful to refine fracture prediction for PMW in this age range, especially when bone microstructure cannot be specifically assessed due to the limited clinical availability of HR-QCT [57–59]. Similarly, a cross-sectional case-control study found that bone microstructure and strength assessment were able to discriminate patients on long-term glucocorticoids with vertebral fracture independently of BMD: noteworthy, with a reduction in total volumetric BMD and cortical thickness at the distal tibia, it was able to identify patients at high risk of vertebral fractures in the subgroup considered to have low fracture risk as assessed by DXA or FRAX^®^ [85].

The main difficulty in the full assessment of bone strength is due to the numerous bone components involved in its definition. The introduction of advanced tools in clinical practice is needed in order to collect additional information for the modelling of bone strength and fracture risk.

### FEA derived from QCT

Several attempts have been performed by FEA derived from QCT [86], sometimes including bone anisotropy information [87], in order to model bone strength and to assess a personalized biomechanical fracture threshold as the ratio of external forces applied to the bone and the FE-computed bone strength [81]. Micro-FEA derived from HR-pQCT was also used in the prospective STRAMBO study, which enrolled 825 men that were followed for 8 years, showing that the distal radius trabecular number was the parameter most strongly associated with fracture risk [88].

### Microindentation

The recently developed in vivo impact microindentation may have a role in the explanation of fracture propensity in case of poor bone quality associated with normal BMD, such as type 2 diabetes, atypical femoral fractures, stress fractures, glucocorticoid treatment and HIV infection [89, 90]. For instance, in a study by Rozental et al. [91], lower bone strength index measured by impact microindentation at the anterior surface of the mid-tibia diaphysis in PMW was associated with distal radius and hip fractures and, if confirmed by prospective trials, might be integrated in models for the identification of women at risk of fragility fractures.

### DXA-based 3D modelling

The most promising future approach could be the integration of the available technologies, such as DXA-based 3D modelling, as shown in a retrospective open-label study aiming to assess the effects of treatments on average changes in volumetric BMD and trabecular architecture over 24 months using DXA-based 3D modelling [92]. Nevertheless, the request for a long time to carry out the FEA hampers its use in the clinical practice, which will perhaps have to wait for the improvement of the underlying technology and mathematical-physical processes.

### Currently unmet clinical needs

Despite the mentioned specific added values of some imaging methods alternative to DXA, the main routinely employed parameter to estimate bone strength and to predict fracture risk is still represented by DXA-measured BMD.

In fact, currently there is not a widely accessible and cost-effective tool capable of significantly improving fracture risk estimation in individuals with osteopenic or normal BMD, which account for about 50% of fragility fractures [93].

Typically, the described approaches increase the duration of the diagnostic procedure and ionizing radiation dose without a clear improvement of the clinical outcome. For instance, a very recent study [94] has evaluated the performance of QCT-based FEA as a predictor of hip fracture risk, documenting that, compared to the traditional DXA, it would imply a 15% increment in the total costs and an over 2000 times higher radiation dose, while reducing the fractured patients by less than 5%. This is also coupled with the important accessibility issues that are common to all the QCT-based techniques, which require all the strictest precautions typical of X-ray-bearing techniques, including also HR-pQCT, the use of which is further hindered by their very limited availability in clinical contexts [57–59].

However, although the cost-effectiveness of osteoporosis prevention through early diagnosis has been demonstrated [95], the identification of the most cost-effective imaging technique through thorough economic analyses (including different fracture states, country-specific aspects, etc.) is still debated in literature [21, 96] and the definition of a specific cost-effectiveness model is beyond the scope of this paper.

Anyway, there is a series of clinical needs that are not effectively satisfied because of the previously mentioned limitations of the currently employed techniques and would require the introduction of reliable methods for bone strength evaluation and fracture risk estimation. The most urgent of these unmet needs include the following: osteoporosis diagnosis in patient categories for which X-ray examinations are not feasible (e.g., paediatric subjects, pregnant and breastfeeding women, etc.); under-diagnosis and late diagnosis; effective bone quality assessment integrated with a reliable fracture risk prediction available in clinical routine; short-term follow-up of patients under treatment, since the present techniques require at least 1 year between two measurements.

## Radiofrequency echographic multi spectrometry (REMS)

### Basic principles

REMS technology is a non-ionizing axial approach for osteoporosis diagnosis. The operating principle is based on the analysis of native raw unfiltered ultrasound signals, the so-called radiofrequency (RF) ultrasound signals, acquired during an echographic scan of lumbar vertebrae and/or femoral neck. The analysis of native unfiltered ultrasound signals allows to retain the maximum information about the characteristics of the investigated tissues, which are normally filtered out during the conventional process of B-mode image reconstruction. The bone health status is assessed through the comparison of the analysed signal spectra with previously derived reference spectral models for the considered pathological and normal conditions [97, 98]. The large amount of collected data related to internal bone structure provides both quantity- and quality-related information, being thus theoretically suitable for the estimation of bone strength and the prediction of fracture risk.

**Fig. 4:**
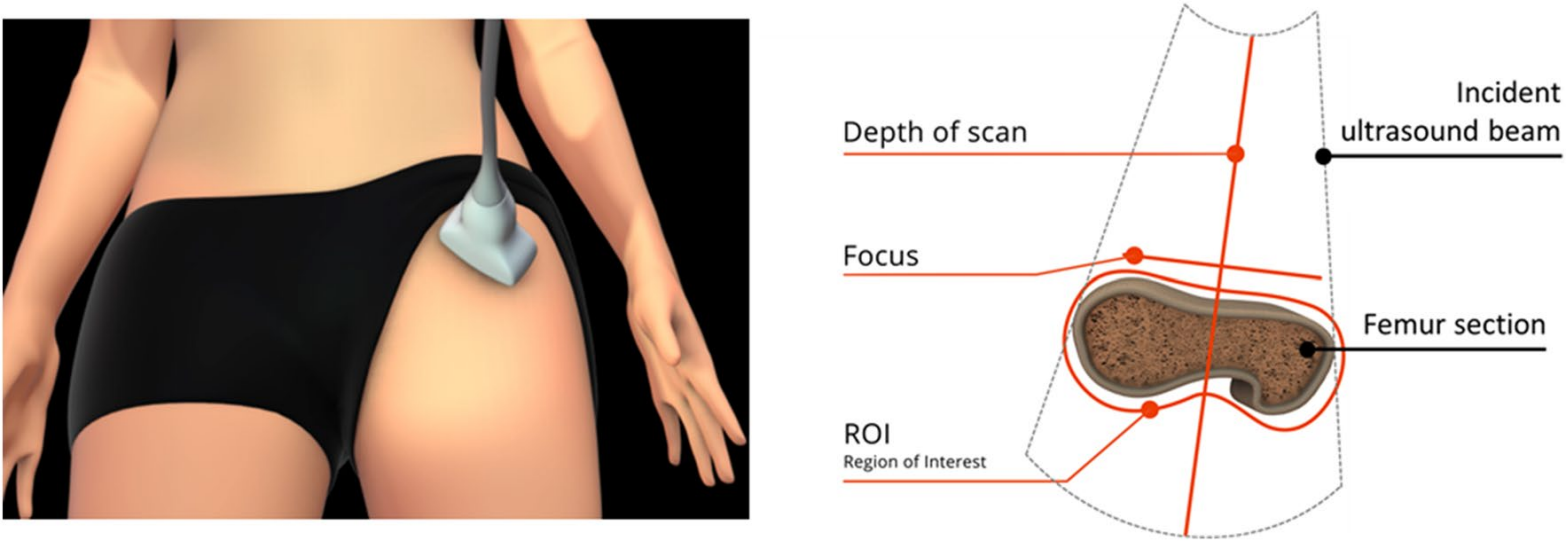
Software-guided REMS acquisition on femoral neck. Before starting the acquisition, the operator sets transducer focus and scan depth in order to visualize the target bone interphase in the central part of the echographic field of view, immediately below the focus position. The software automatically detects the bone interface and identifies the region of interest (ROI)

**Fig. 5:**
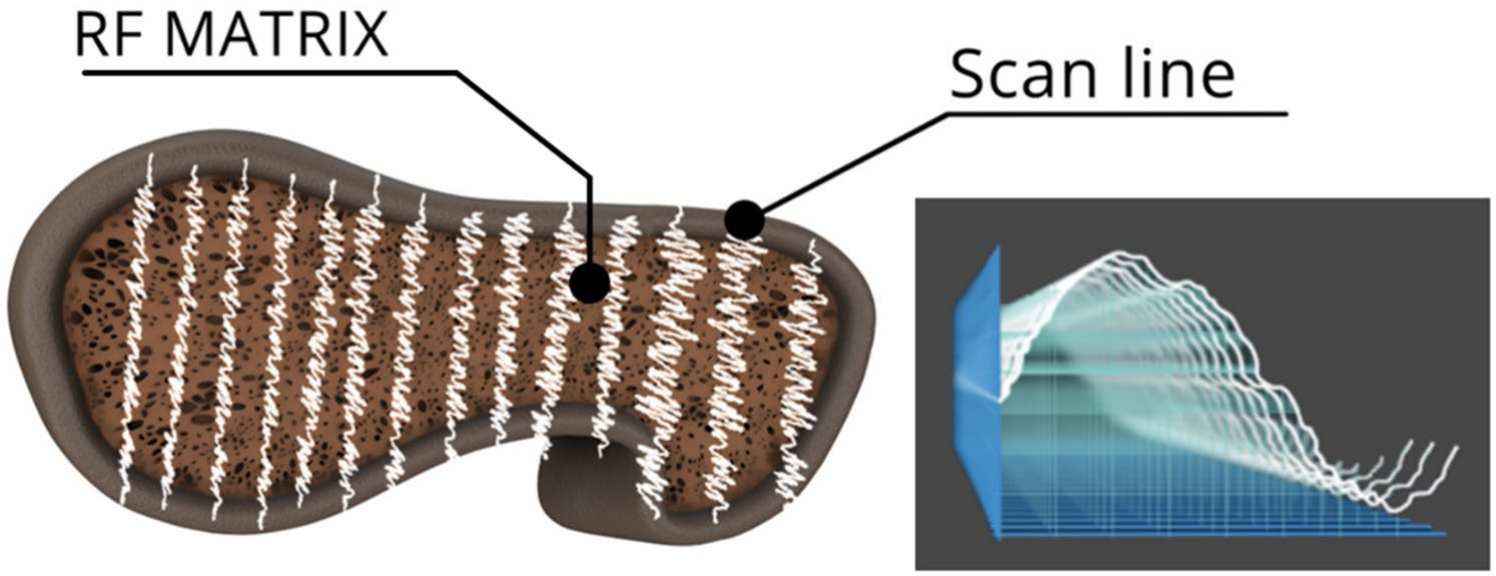
REMS analysis is characterised by the parallel processing of the native raw unfiltered signals of several scan lines, deriving one spectrum from each scan line (sample spectra are shown on the right)

In more detail, in a REMS investigation, the probe is placed on the abdomen or on the hip in order to visualize of the target bone interface and the operator has to set the appropriate values of scan depth and transducer focus. Subsequently, the software detects the sought bone interfaces in the sequence of acquired frames and identifies the regions of interest (ROIs) for the diagnostic evaluation (Fig. 4). One key-feature lies in the exploitation of B-mode images for the identification of target bone interfaces and related ROIs, combined with the diagnostic analyses performed on the RF data. REMS analysis is then characterised by the parallel processing of the unfiltered signals of several scan lines (Fig. 5).

The analysis of single scan line spectra allows the automatic exclusion of signals corresponding to artefacts, such as calcifications or osteophytes, thanks to the identification of unexpected spectral features.

**Fig. 6:**
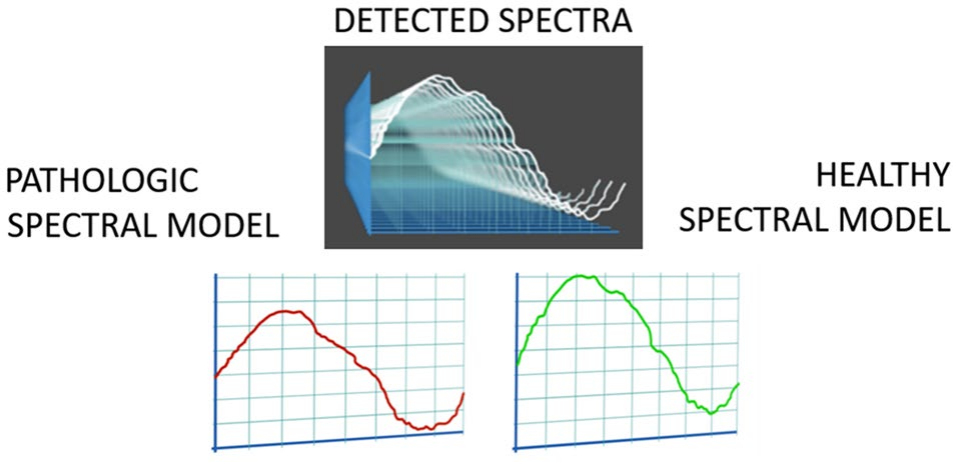
Patient-specific spectra undergo advanced comparisons with age-, sex-, BMI- and site-matched spectral models of pathologic and healthy conditions

The selected measured data are finally synthetized in a patient-specific spectrum of the considered bone target, which undergoes an advanced comparison with gender-, age-, site- and BMI-matched reference spectral models extracted from a dedicated database (Fig. 6).

Actually, the spectral modifications introduced by the physical properties of the bone structure that has backscattered the ultrasound signals are identified by the comparison procedure, resulting in a BMD estimation and in the consequent diagnostic classification as healthy, osteopenic or osteoporotic. The adopted approach is based on the calculation of the Osteoporosis Score, which corresponds to the percentage of analysed spectra that were classified as “osteoporotic” through the dedicated spectral analyses [97, 98]. Linear equations are then employed to transform Osteoporosis Score into BMD values.

REMS approach has the potential to calculate also parameters different from BMD, derived from bone quality indicators and further related to bone strength. Among these, Fragility Score (FS) has been developed as an independent indicator of bone quality that provides an estimation of fracture risk independently of BMD. It is a dimensionless number in the range 0–100 obtained from the comparison between the patient-specific spectrum and reference spectral models obtained from patients with and without osteoporotic fractures. Recent preliminary studies have shown that FS has a good accuracy in the discrimination between fractured and non-fractured subjects, and also that FS is significantly correlated with the fracture risk calculated by FRAX^®^ when the latter included also the results of the femoral neck densitometry [99–101].

Ongoing developments of REMS are addressed to the investigation of other musculoskeletal tissues, such as muscles and cartilage, in order to monitor the onset and evolution of diseases like sarcopenia and arthrosis by early detecting the corresponding tissue deteriorations through dedicated REMS parameters.

### Clinical validation studies

The application of REMS technology for osteoporosis diagnosis has been clinically validated through an observational multicentre clinical trial involving 7 Italian centres that enrolled over 1900 PMW [102]. The aim of the study was to assess the precision and diagnostic accuracy of REMS in comparison with the clinical gold standard reference, represented by DXA. All DXA acquisitions were double-checked to avoid possible errors as identified by Messina et al. [103]. Similarly, a quality control was performed on all the REMS reports in order to verify the correct selection of scan depth and transducer focus. The analysis of the temporal distribution of the errors indicated the possibility of improving the clinical practicability of REMS through a more rigorous training of the operators, aimed at reducing the time span of the learning curves and the related initial error rates. After the cross-check, 1195 spinal and 1373 femoral comparable cases were analysed.

The REMS intra-operator precision, expressed as root-mean-square coefficient of variation (RMS-CV) was 0.38% (95% confidence interval: 0.28–0.48%) for lumbar spine and 0.32% (0.24–0.40%) for femoral neck. The corresponding least significant change for the 95% confidence level, calculated via the ISCD precision calculator (available at http://www.iscd.org/resources/calculators/), was 1.05% for lumbar spine and 0.88% for femoral neck. The inter-operator repeatability was 0.54% for spine and 0.48% for femoral neck.

Afterwards, diagnostic accuracy of REMS was assessed by determining the concordance of REMS and DXA in the discrimination between osteoporotic, osteopenic and healthy patients through the calculation of the corresponding Cohen’s kappa (*k*), obtaining *k *= 0.82 for lumbar spine and *k *= 0.79 for femoral neck.

Sensitivity of REMS in discriminating osteoporotic subjects from non-osteoporotic ones turned out to be 91.7% for spine and 91.5% for femur, while specificity was 92.0% and 91.8%, respectively. Furthermore, the degree of correlation between DXA and REMS *T*-score values resulted to be *r *= 0.94 for lumbar spine and *r *= 0.93 for femoral neck (*p* < 0.001 for both).

Moreover, the agreement between BMD values obtained by DXA and REMS was calculated through the Bland–Altman method [104]: the average difference (expressed as bias ± 2 SDs) was − 0.004 ± 0.088 g/cm^2^ for lumbar spine and − 0.006 ± 0.076 g/cm^2^ for femoral neck. The standard error of the estimate (SEE) resulted equal to 0.044 g/cm^2^ (5.3%) for lumbar spine and 0.038 g/cm^2^ (5.8%) for femoral neck.

The results obtained from the referred multicentre clinical study showed that REMS technology has a high sensitivity and specificity in osteoporotic patient identification, with a significant diagnostic agreement with the gold standard DXA in the classification of patients as healthy, osteopenic or osteoporotic (total diagnostic concordance was 88.8% for lumbar spine and 88.2% for femoral neck). Both intra- and inter-operator variability associated with REMS investigations were better than the corresponding values typically reported in literature for the employed comparative gold standard DXA [105, 106].

Thanks to its radiation-free approach, REMS might be applied for population mass investigations or prevention programs, early diagnosis in clinical practice and therapeutic short-term follow-up.

In the meantime, in October 2018 the first device implementing the REMS approach has received the clearance of the U.S.A. Food and Drug Administration (FDA) for the measurement of the diagnostic parameters BMD, *T*-score and *Z*-score, and for monitoring bone changes in the clinical routine.

### Further studies and clinical perspectives

An International Multicentre Clinical Trial, called “Echo-Bone” and focused on an extended study of the REMS approach for osteoporosis diagnosis in a wide European clinical context through a head-to-head trial with DXA, is currently ongoing in five reference centres for osteoporosis management. At an interim analysis on 595 patients enrolled in Barcelona (Spain) [107], preliminary data confirm the recently published results [102].

Then, another recently completed longitudinal study [108] has quantified the predictive value of REMS *T*-score in identifying patients at risk for osteoporotic fractures by performing a follow-up of up to 5 years for the possible occurrence of fragility fractures in a population of 1370 women who had undergone baseline spinal REMS and DXA scans. After the follow-up period, all the subjects were divided into two Groups: Group A (with incident fractures) and Group B (without incident fractures). As expected, significant differences between the two groups were found in both REMS *T*-score (− 2.68 ± 1.28 in Group A vs − 2.03 ± 1.23 in Group B, *p *< 0.001) and DXA *T*-score (− 2.52 ± 1.20 in Group A vs − 2.08 ± 1.17 in Group B, *p *< 0.001). The employment of the typical threshold of *T*-score ≤ − 2.5 as a cut-off value for both the techniques produced the following results in the identification of Group A patients by the two techniques: sensitivity = 65% and specificity = 57% for REMS (OR = 2.6); sensitivity = 57% and specificity = 57% for DXA (OR = 1.7). This performance of REMS *T*-score in the identification of patients at high risk for osteoporotic fractures will have to be further verified on a wider population through the outcome of a similar study that is currently ongoing in six clinical centres.

In general, the available data suggest that REMS technology might have a beneficial impact on current diagnostic protocols and subsequent patient management in the clinical routine. Further clinical scenarios are envisaged for additional REMS applications, including in particular fracture risk assessment in paediatric patients and pregnant women, and also in patients at risk of secondary osteoporosis (e.g., diabetic, nephropathic, oncological patients).

REMS adoption in the clinical routine is expected to increase in the near future because of the mentioned evidences and advantages, and also because the technology is currently undergoing the evaluation procedures to be included in the relevant international guidelines. Noteworthy, this technology does not require radiological protection, which might have been a problem for the reception in primary care in some countries. Finally, the portability facilitates the employment on hospitalized fractured patients not-transferrable to the densitometry units and on patient follow-up at home.

## Conclusions

The impact of osteoporosis and the resulting bone fracture on both patient’s life and healthcare systems is constantly increasing: means for early diagnosis and monitoring of this disease are urgently needed in order to prevent and reduce the occurrence of fractures. BMD measurement based on DXA remains the current gold standard for osteoporosis classification, as defined by WHO. However, the above-mentioned clinical situation and the reported limitations of this technique boosted the scientific research to identify reliable and accurate alternative approaches for bone strength estimation and identification of individuals at high risk of fracture. Those efforts are leading to a deeper understanding of intrinsic bone characteristics, related not only to bone quantity but also to bone quality, which might contribute to improve the accuracy of bone strength measurement and fracture risk assessment.

In this context, REMS represents the first clinically available method for direct non-ionizing measurement of lumbar and femoral BMD. Available scientific evidences describe REMS-estimated BMD as an accurate diagnostic parameter, which resulted also a predictor of incident clinical fracture risk in a representative sample of female subjects. Moreover, REMS has shown a further potential in the assessment of skeletal fragility based on bone structure quality through the Fragility Score parameter, which is independent from the densitometric evaluation.
